# Flexural Performance and End Debonding Prediction of NSM Carbon FRP-Strengthened Reinforced Concrete Beams under Different Service Temperatures

**DOI:** 10.3390/polym15040851

**Published:** 2023-02-08

**Authors:** Marta Baena, Younes Jahani, Lluís Torres, Cristina Barris, Ricardo Perera

**Affiliations:** 1 Analysis and Advanced Materials for Structural Design (AMADE), Polytechnic School, University of Girona, 17003 Girona, Spain; 2 Department of Mechanical Engineering, Technical University of Madrid, 28006 Madrid, Spain

**Keywords:** NSM CFRP-strengthening, end debonding, service temperature, experimental

## Abstract

This paper aims to evaluate the influence of relatively high service temperatures (near or beyond the glass transition temperature (*T_g_*) of epoxy adhesive) on the flexural performance and end debonding phenomenon in near-surface mounted (NSM) carbon fiber-reinforced polymer (CFRP)-strengthened, reinforced concrete (RC) beams. To this end, an experimental program consisting of 24 beams (divided into four groups) was performed, where different parameters was combined (i.e., service temperature, steel reinforcement ratio, CFRP ratio, and concrete compressive strength). In addition, the effect of the testing temperature on the end debonding phenomenon was investigated with an analytical procedure according to *fib* Bulletin 90, and the predictions were compared to experimental results. Taking specimens tested at 20 °C as a reference, no considerable change was observed in the ultimate load of the specimens tested below 60 °C (being in the range of epoxy *T_g_*), and all specimens failed by FRP rupture. On the other hand, the increase in testing temperature up to 70 and 85 °C was followed by a decrease in the capacity of the strengthened beams and a change in failure mode, moving from FRP rupture to end debonding and concrete crushing. The analytical procedure successfully predicted the occurrence of premature end debonding failure and demonstrated that the effect of temperature on the mechanical properties of materials can be a key factor when predicting the premature end debonding in a NSM joint.

## 1. Introduction

Fiber-reinforced polymers (FRPs) have become popular in the construction industry, specifically in repairing and strengthening aged, damaged, or overloaded concrete structures (beams, slabs, columns, etc.). To this end, different techniques are used in FRP-strengthened structures, namely externally bonded reinforcement (EBR) and near-surface mounted (NSM) techniques [1,2,3]. In the EBR technique, the surface of the concrete must be treated and then the laminate should be attached to the surface of the member using an adhesive. In the NSM technique, grooves are cut into the concrete surface and then the FRPs are inserted into these grooves using a proper adhesive. When compared to the EBR system, the NSM technique has several advantages such as: no need for surface treatment, less susceptible to environmental conditions, less prone to vandalism, and good finished surface, among others [1,2,3].

In FRP-strengthened reinforced concrete (RC) beams, three different materials are involved to transfer the shear load between the concrete and FRP, namely FRP, bonding material (typically epoxy/cement-based adhesive), and concrete. Therefore, the performance of the FRP-strengthened RC structure is not only dependent on the interaction between these materials but also on the individual properties of each material, and how they are affected by external factors. Among these materials, monitoring the performance of the epoxy adhesive is very important due to the influence of the environmental conditions, especially temperature changes near or beyond the glass-transition temperature (*T_g_*), on the mechanical properties of epoxy adhesives [4,5,6,7,8]. Because of this, the service temperature for epoxy-bonded joints has been limited to avoid possible degradation in the efficiency of the strengthening system. As an example, ACI 440.2R-17 [9] proposes to limit the maximum service temperature of the strengthening system to be 15 °C below the epoxy *T_g_*. Furthermore, according to *fib* Bulletin 90 [10], this service temperature should be 20 °C less than the *T_g_*, to avoid any considerable change in the adhesive properties. In addition to design codes, different studies have been performed to define the service temperature in FRP-strengthened systems [11,12,13,14,15]. Klamer [11] suggested the service temperature to be limited to 10 °C less than the *T_g_* of the adhesive for Externally Bonded Reinforcement (EBR) systems. Michels et al. [12] summarized the various design code provisions and defined the service temperature in FRP-strengthened RC structures to be 10 to 20 °C less than *T_g_*. To avoid the effect of temperature in the creep of epoxies, Ferrier et al. [13] limited the service temperature to be 15 °C less than the *T_g_*. Moreover, in similar studies [14,15], and with the aim at avoiding premature debonding of FRP sheets from the concrete surface, the maximum temperature was limited to be 10 °C less than the *T_g_*. These limitations are related to the EBR system, while for the NSM technique no specific limitation has been stated.

It is important to mention that extensive research has been performed on the effect of elevated temperatures on the performance of an FRP–concrete joint, as well as on the behavior of the constituent materials [16,17]. However, when evaluating the behavior of the joint, most of the existing works deal with single and double lap shear tests, and less literature exists to evaluate the effect of elevated temperatures on the flexural performance of FRP-strengthened RC beams. According to the existing literature for the EBR system, Klamer et al. [18] studied the effect of three different temperatures (i.e., 20, 50, and 70 °C) on flexural performance of four different EBR-strengthened RC beams. The *T_g_* of the epoxy was declared to be 62 °C. The results showed similar failure loads for those specimens with long bonded length, whilst specimens with short bond length exposed to 70 °C suffered a reduction in the ultimate load and a change in failure mode from concrete–adhesive interface failure to concrete cover rip-off. In another study, Krzywoń [19] investigated the effect of different temperatures (20 to 80 °C) on EBR-strengthened beams (*T_g_* of the epoxy was 45 °C). The results showed that the ultimate load remained mostly unchanged for testing temperatures below 62 °C, and it was reduced by 20% for testing temperatures beyond 70 °C. This reduction in load capacity was followed by the failure mode changing from cohesive in concrete to adhesive at the concrete–epoxy interface. 

Moving from the EBR system to NSM, many studies have been done to evaluate the flexural performance of NSM FRP-strengthened RC beams at room temperature [20,21,22,23,24,25,26] or fire conditions [27,28,29,30,31,32], but evaluation of their behavior at service temperatures near or beyond the *T_g_* of epoxy is almost missing in the literature. Silva et al. [33] studied the effect of service temperatures (up to 80 °C) on the flexural performance of NSM-strengthened concrete slabs with epoxy adhesive having a *T_g_* equal to 55 °C. According to the experimental results, the maximum load capacity was observed in the slab at 40 °C, which was attributed to a possible post-curing effect in the epoxy resin. With the increase in the temperature up to 80 °C, the capacity of the slab decreased by 12% and the failure mode changed from concrete crushing to cohesive failure at the epoxy. In a more recent study, Jahani et al. [34] investigated the flexural performance of NSM-strengthened RC beams subjected to various temperatures (20 to 85 °C), and observed that temperature affected the load capacity and failure mode of those specimens tested at higher temperatures. 

Based on the scarce literature on the effect of high service temperatures on the flexural performance of NSM carbon FRP (CFRP)-strengthened beams, and its consequences on possible end debonding phenomena, this paper collects and presents experimental results on 24 beams tested by the authors, so that different steel reinforcement ratios, service temperatures and concrete grades (compressive strength of concrete) are considered. In this sense, the results are presented and analyzed in terms of load–deflection curves and failure modes. Additionally, the experimental results are compared to analytical predictions on end debonding in which the effect of temperature on epoxy and concrete mechanical properties are included.

## 2. Experimental Program

### 2.1. Specimens and Test Configuration

The test program consisted of a total of 24 specimens divided into four groups, as presented in Table 1. To evaluate the efficiency of the CFRP-strengthening, four parameters were considered (i.e., steel reinforcement ratio, CFRP ratio, testing temperature, and concrete compressive strength). Group 1 included eight specimens with a concrete compressive strength equal to 31.8 Mpa and a steel reinforcement ratio of 1.14%, distributed as follows: two control beams subjected to two different temperatures (20 and 40 °C) and six strengthened beams with three different CFRP ratios (0.06%, 0.12%, and 0.18%, corresponding to a CFRP area equal to 14, 28, and 42 mm^2^, respectively) subjected to 20 and 40 °C. In Group 2, six specimens were tested to evaluate the effect of higher temperatures. To this end, two control beams with a steel reinforcement ratio of 1.14% (at 20 and 70 °C) and four strengthened beams with two CFRP strips (CFRP area of 28 mm^2^, corresponding to a CFRP ratio equal to 0.12%) at various temperatures (20, 60, 70, and 85 °C) were cast with a concrete compressive strength of 40.8 Mpa. The specimens in Groups 3 and 4 had a concrete compressive strength of 48.1 Mpa but two different steel reinforcement ratios: *ρ* = 0.79% and 1.14% for Group 3 and 4, respectively. Group 3 included two control beams and four strengthened beams with two different CFRP ratios (0.06% and 0.18%, corresponding to a CFRP area equal to 14 and 42 mm^2^, respectively) subjected to 20 and 50 °C, thus making a total of 6 beams. Finally, a total of four specimens in Group 4 were distributed as two control beams and two strengthened beams with a CFRP ratio of 0.18% (CFRP area equal to 42 mm^2^) and subjected to 20 and 50 °C. 

The specimens’ designations are in the form X-Y-Z, where X denotes the type of beam (CB meaning control beam, and SB1S, SB2S, and SB3S referring to strengthened beams with one, two, and three CFRP strips, respectively); Y indicates the number of the Group that the specimen belongs to; And Z stands for the testing temperature. For instance, CB-1-40, refers to the control beam in Group 1 tested at 40 °C. Furthermore, SB3S-3-50 refers to a beam strengthened with three CFRP strips from Group 3 and tested at 50 °C. It should be mentioned that reference to the CFRP ratio in the specimens’ designations is done based on the number of CFRP strips included in the cross-section. However, to have a clearer idea on the amount of CFRP strengthening included in each beam, the CFRP ratio is also presented in Table 1. To derive it, we considered the area of one CFRP strip as 14 mm^2^, and the dimensions on the cross-section of the beam were kept constant along the whole experimental campaign.

**Figure 1:**
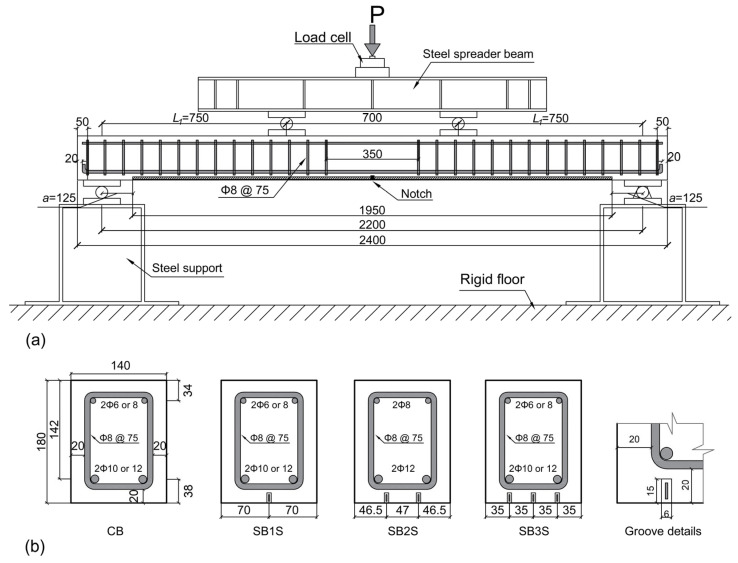
Details of the tested beams. (**a**) Test setup (*L_1_* is the shear span of the beam and *a* is the distance between support and end of CFRP strip); (**b**) beam sections (dimensions in mm).

The beams were tested under a four-point bending configuration (see Figure 1). They had a total length of 2400 mm and a clear span length equal to 2200 mm, with a cross-section of 180 mm × 140 mm. The loading span and shear span were 700 mm and 750 mm, respectively. In Groups 1, 2, and 4, two ribbed steel bars with a diameter of 12 mm were used in the tension side of the beams and two ribbed steel bars with a diameter of 8 mm were used in the compression side of the beams. In Group 3, two ribbed steel bars with a diameter of 10 mm and two ribbed steel bars with a diameter of 6 mm were used in the tension and in the compression side of the beams, respectively. To avoid shear failure, a shear reinforcement with a diameter of 8 mm and distance equal to 75 mm was used in all beams. For strengthened beams, CFRP strips with a cross-section of 1.4 mm × 10 mm and bonded length of 1950 mm were used. To allocate the CFRP strips, grooves with dimensions of 6 mm × 15 mm were cut in the soffit of the beam. The dimensions of the grooves and distance between them were set based on *fib* Bulletin 90 [10] recommendations. To initiate a crack at specific position, all beams had a 5 mm wide and 15 mm deep notch at midspan. The flexural test was performed under displacement control at a rate of 0.6 mm/min. 

### 2.2. Instrumentation

**Figure 2:**
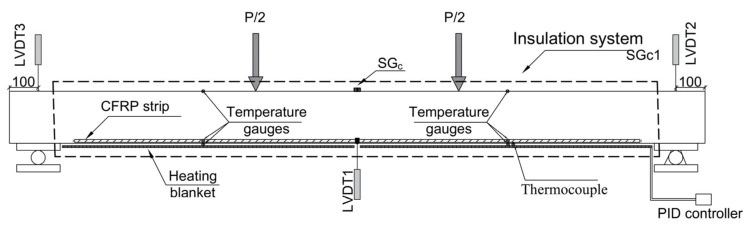
Beam instrumentation: linear vertical displacement transducers (LVDTs), position of concrete strain gauge, temperature gauges, heating system, and insulation system.

Figure 2 shows the instrumentation used in this experimental work. A linear vertical displacement transducer (LVDT) with 100 mm stroke (with linearity error of ±0.10% F.S.) was used in the mid-section of the beam to measure the central deflection (LVDT1). Moreover, to measure the support settlement in all the tests (strengthened and unstrengthened beams), two LVDTs with 25 mm stroke (with linearity error of ±0.10% F.S.) were used in both supports (LVDT2 and LVDT3). To register the strain variation during loading process, one strain gauge was installed on the surface of the concrete (SG_c_) as shown in Figure 2. 

Prior to testing, the beams were heated up to the target temperature using heating blankets that were attached to the soffit of the beam. A proportional integral derivative (PID) controller was utilized for the heating process, and Type-T thermocouples, located between the heating blanket and the soffit of the beam, were used as temperature controller sensors. An isolation system (rock wool with aluminum foil) was used to speed the heating process and to ensure a better heat distribution. To record and monitor the temperature variation during the heating process and flexural testing, different temperature gauges were glued on the concrete surface at the top and bottom of the beam, on the surface of the CFRP, and on the surface of the epoxy adhesive (see Figure 2).

**Figure 3:**
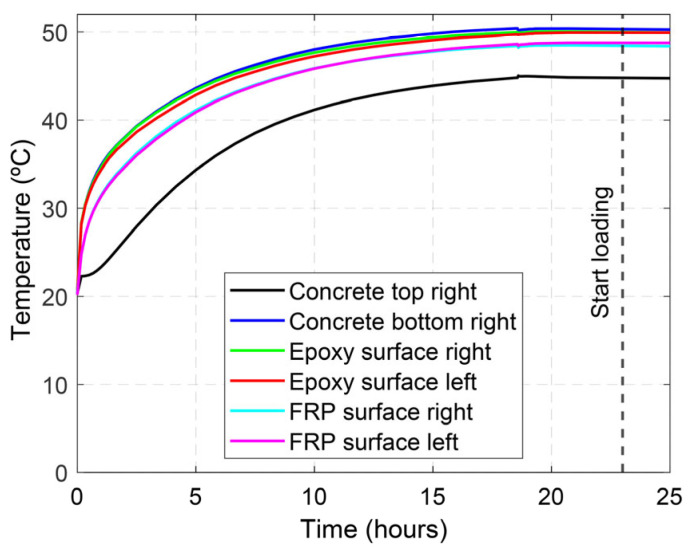
Representative evolution of temperature at different locations within the heating process of a specimen tested at 50 °C.

**Figure 4:**
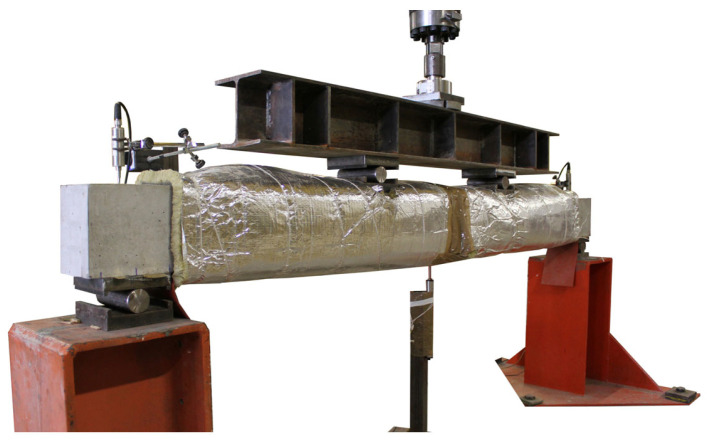
General view of experimental setup.

It should be mentioned that the temperature gauges glued on the surface of the CFRP strip were installed prior to introducing the CFRP strip into the groove, and a thin protection layer was applied to protect them from the wet environment during the curing of the epoxy adhesive. Moreover, temperature gauges glued on the surface of the epoxy adhesive were installed once the epoxy was cured, at the outer part of the section. The tests started when the average temperature in the soffit of the beam was stabilized, almost 24 h after the heating process started, and it was kept constant during the loading (see Figure 3). A general view of the test setup is shown in Figure 4.

### 2.3. Materials

#### 2.3.1. Concrete

Three different batches of concrete were used. The details of the concrete batches are shown in Table 2. To improve the workability of the concrete, a viscosity modifier and underwater admixture were used. The experimental compressive strength (*f_c_*), tensile strength (*f_t_*), and modulus of elasticity (*E_c_*) of the concrete were determined from three cylinder specimens (300 mm nominal height and 150 mm nominal diameter), according to UNE-EN 12390-3 [35], UNE-EN 12390-6 [36], and ASTM C469 [37] standards, respectively.

#### 2.3.2. Steel Reinforcement

The mechanical properties of the steel bars were obtained from tension tests based on UNE-EN ISO 15630-1 [38]. The yielding stress (*f_y_*), the ultimate stress (*f_u_*), and the modulus of elasticity (*E_s_*) for each group of specimens are shown in Table 3.

#### 2.3.3. CFRP

CFRP strips, consisting of unidirectional carbon fibers (with a volume content fiber higher than 68%) held together by an epoxy resin, were used to strengthen the specimens [39]. The CFRP strips had a cross-section of 1.4 mm × 10 mm, and their tensile mechanical properties were obtained according to ISO 527-5 [40] recommendations. An ultimate tensile strength (*f_u,FRP_*) of 2251.4 Mpa (CoV = 3.2%), an ultimate tensile strain (*ε_u,FRP_*) of 0.0133 (CoV = 7.2%), and a modulus of elasticity (*E_FRP_*) of 169.5 Gpa (CoV = 6.3%) were obtained [34].

#### 2.3.4. Epoxy Adhesive

The adhesive used in this study is a high performance, solvent-free, thixotropic, and grey two-component epoxy adhesive specially developed for bonding CFRP to concrete under the commercial name of S&P220HP. According to the manufacturer’s product data sheet [41], the components A (resin) and B (hardener) should be mixed at a ratio of 2:1 by weight, and the suggested curing duration is 7 days. However, in this work, an average curing of 12 days was used for the epoxy adhesive. The glass-transition temperature (*T_g_*) of the epoxy was determined based on two well-known available methods, namely differential scanning calorimetry (DSC) (ASTM E1356 [42]) and dynamic mechanical analysis (DMA) (ASTM D5023 [43]). According to test results, the *T_g_* of the epoxy was in the range of 53.9–65.3 °C [34]. 

The tensile properties of the epoxy adhesive were determined by dog-bone specimens following ISO-527-1 [44] specifications. In addition, the compressive strength of the epoxy adhesive was determined according to EN 196-1 [45] using prism specimens. Of the three components of the bonded joint (CFRP, epoxy adhesive, and concrete), the epoxy adhesive is the most likely to be influenced by variations in testing temperature. Therefore, to evaluate the effect of temperature on the mechanical properties of the epoxy adhesive, characterization tests were performed at different temperatures. For that purpose, the specimens (three samples for each temperature) were placed in a thermal chamber (mounted onto the testing machine) 24 h prior to testing and, after stabilizing the temperature inside the thermal chamber, the specimens were loaded until failure. The temperature was kept constant before and during the test. The results of the tension and compression tests are shown in Table 4 [8]. 

## 3. Results and Analysis

### 3.1. Experimental Load–Deflection Curves

**Figure 5:**
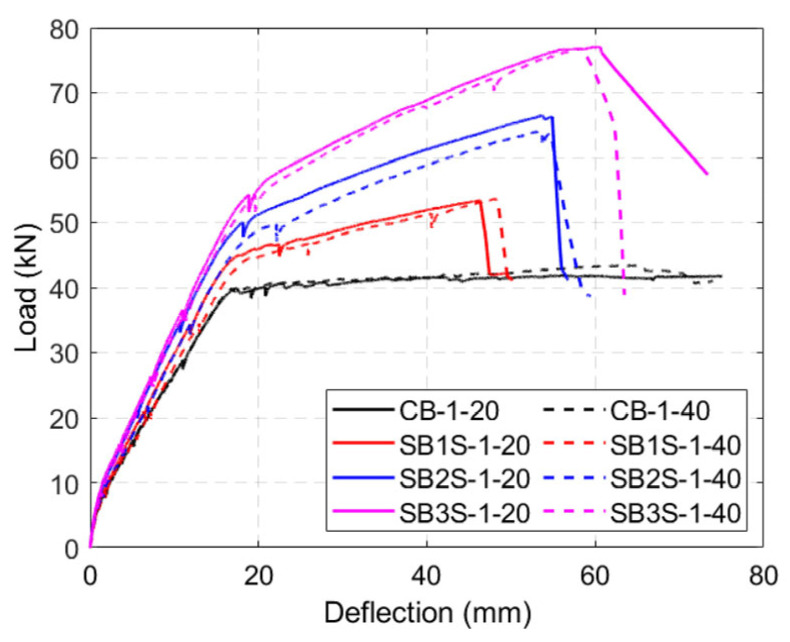
Load–deflection curves of specimens in Group 1.

**Figure 6:**
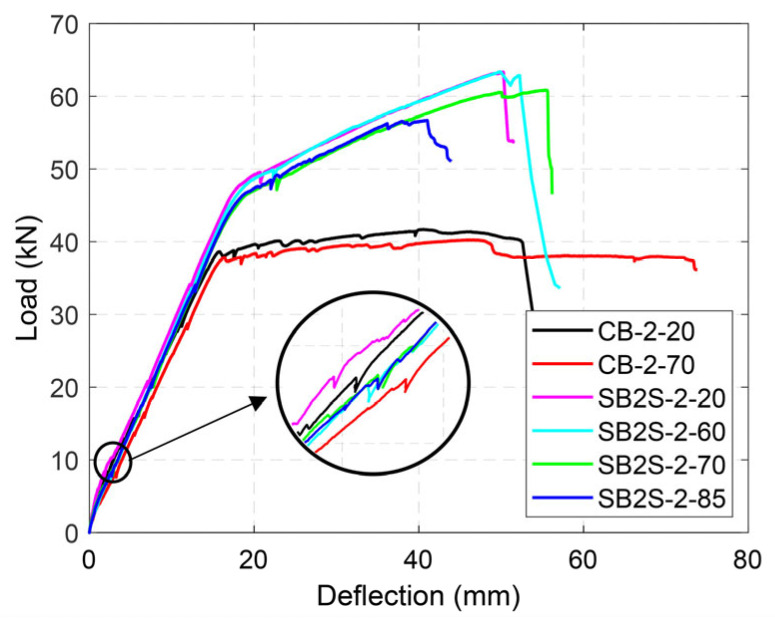
Load–deflection curves of specimens in Group 2.

**Figure 7:**
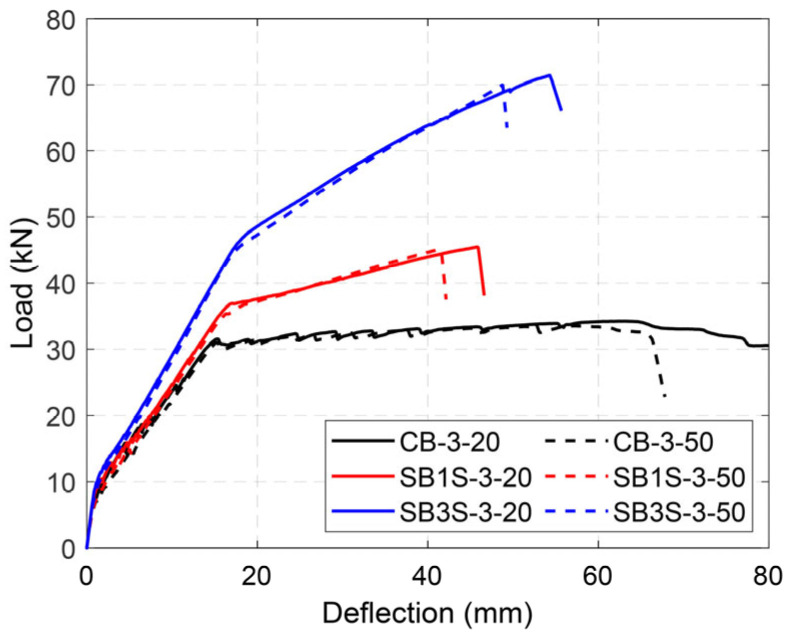
Load–deflection curves of specimens in Group 3.

**Figure 8:**
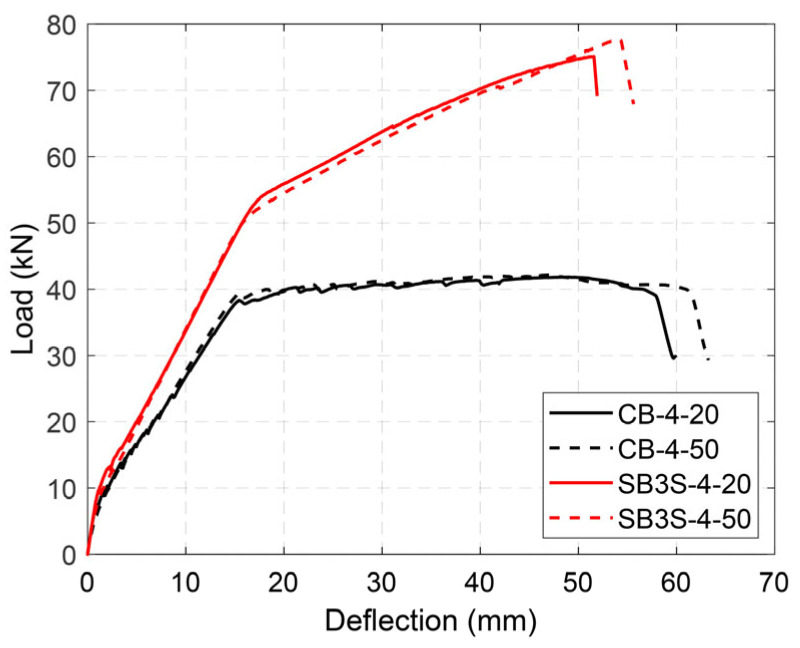
Load–deflection curves of specimens in Group 4.

The load–deflection curves of the tested beams are shown in Figure 5, Figure 6, Figure 7 and Figure 8 for Groups 1 to 4, respectively. Load–deflection curves of the unstrengthened and strengthened beams followed a trilinear diagram having the following phases: (i) the initial uncracked elastic phase; (ii) cracked phase up to steel yielding; and (iii) post-yielding phase up to failure. The unstrengthened beams behaved in a perfectly plastic manner in the third phase, whereas the strengthened beams indicated continuous hardening up to maximum load capacity [3]. As a general observation, larger CFRP ratios (i.e., the existence of a larger amount of CFRP material) produced larger yielding loads and ultimate loads, irrespective of the applied temperature, as expected. It should be mentioned that due to higher shrinkage measured in the concrete for specimens in Group 2 [34], the cracking load was relatively lower than the expected one. The experimental results are summarized in Table 5.

According to Figure 5 that refers to specimens in Group 1, the increase in the testing temperature from 20 °C to 40 °C did not significantly affect the load–deflection curves of the specimens, except for a very slight reduction on specimen’s stiffness. 

Figure 6 shows the effects of relatively high temperatures in the load–deflection curves of beams belonging to Group 2. In this group, the steel reinforcement ratio and CFRP ratio were kept constant, while the temperature was increased up to near or beyond the *T_g_* of the epoxy (i.e., 60, 70, and 85 °C). Similar to the results obtained for Group 1, increasing the testing temperature up to 60 °C did not significantly affect the ultimate load (i.e., capacity) of the strengthened beams. On the contrary, strengthened specimens tested at temperatures equal to 70 °C and 85 °C showed some reduction in their ultimate load equal to 4.0% and 10.5%, respectively, compared to the corresponding value of the strengthened beam tested at 20 °C (beam SB2S-2-20). Moreover, due to effect of temperature in the mechanical properties of the concrete, ultimate load in the control beam was also reduced (by 3.5%) when the specimen was subjected to 70 °C. 

To evaluate the effect of the steel reinforcement ratio and testing temperature just below the *T_g_* of the epoxy adhesive, the results from the specimens belonging to Groups 3 and 4 were analyzed. Figure 7 and Figure 8 show the load–deflection curves of the specimens in Groups 3 and 4, respectively. When the steel reinforcement ratio changed from 0.79% to 1.14%, an increase in the yielding and ultimate loads was observed. Furthermore, as was expected and observed in the other groups, any increase in the CFRP ratio resulted in an increase in the load capacity of the system. It should be mentioned that the benefits of the CFRP strengthening system were larger in beams with lower steel reinforcement ratios, as depicted by the comparison between the strength increase ratios of the specimens in Groups 3 and 4 (see Table 5).

Similar to the specimens in Group 1, the increase in the testing temperature up to 50 °C had no effect in the response of the specimens in Groups 3 and 4. According to the experimental results (in all groups), it can be concluded that the NSM strengthening system is slightly susceptible to temperature variations, as applying temperatures up to 60 °C (in the range of epoxy *T_g_*) had no considerable effect. Therefore, the results suggest that the design service temperature limitation for NSM technique could be increased to values closer to the epoxy *T_g_*. 

### 3.2. Failure Modes

**Figure 9:**
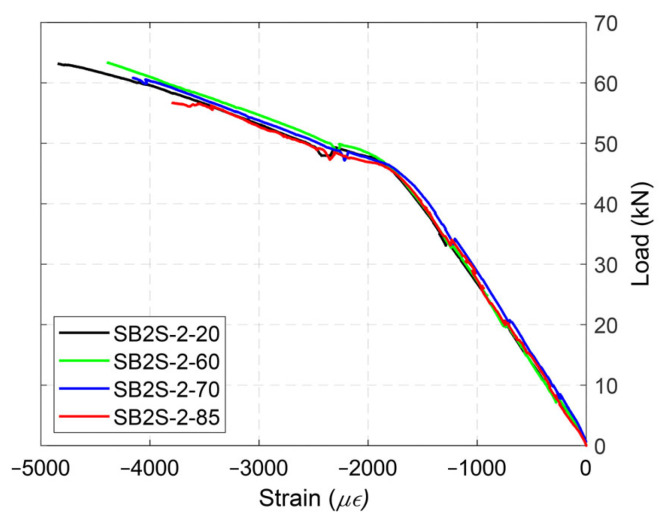
Concrete strain evolution for the strengthened specimens in Group 2.

The failure mode in all the control (unstrengthened) beams was concrete crushing after yielding of the steel reinforcement (CC), irrespective of the testing temperature. Strengthened specimens belonging to Groups 1, 3, and 4 experienced FRP rupture (FR), and the failure mode did not change with the increase in the testing temperature. Similar behaviors were observed by other authors [18,19,33] and may be attributed to the fact that the temperature applied to those beams was far below the *T_g_* of the epoxy adhesive (experimentally determined to be in the range of 53.9 °C < *T_g_* < 65.3 °C) and the epoxy may have post-cured [8,33,34]. Finally, different failure modes were observed in strengthened specimens belonging to Group 2, depending on the temperature. Specimens tested under 20 °C and 60 °C failed by FRP rupture (FR). With an increase in the temperature to 70 °C, and due to a reduction in the bond resistance between the epoxy and concrete, the beam failed by end debonding (ED) before reaching the ultimate capacity of the strengthening system. A small disturbance in the load–deflection curve at a load level around 60 kN, close to the failure load, might indicate the initiation of the debonding (see Figure 6). Finally, in the specimen tested at 85 °C (specimen SB2S-2-85), a reduction of the stiffness of the specimen was observed around 56 kN, and the load–deflection curve started to flatten, approaching a plateau, which may be due to the achievement of the capacity of the “heated” bonded joint. Although the bonded joint was damaged, the beam eventually failed by concrete crushing (CC). It should be mentioned that at that level of load (56 kN), typical values for the ultimate strain under compression for concrete (around 3.5 and 4%) were registered, as shown in Figure 9 (strains measured with strain gauge SG_c_ in Figure 2). 

**Figure 10:**
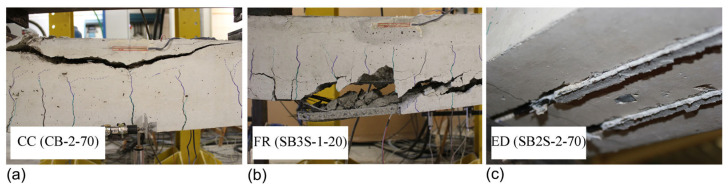
Representative failure modes of the specimens. (**a**) CC (concrete crushing); (**b**) FR (FRP rupture); and (**c**) ED (end debonding).

Representative images of the different failure modes are shown in Figure 10.

According to experimental observations, up to 60 °C, all strengthened beams failed by FRP rupture without any premature end debonding, which indicates the good performance of the NSM strengthening system for the chosen beam dimensions and setup configuration. Therefore, unlike recommendations for service temperatures of the EBR system (e.g., the *fib* bulletin 90 [10]), the efficiency of the NSM system was not affected by temperatures in the range of the *T_g_* of epoxy (i.e., 60 °C).

### 3.3. End Debonding Prediction

End debonding is one of the most common failure modes in NSM strengthening techniques, especially in the case of short anchorage length [46]. Therefore, the adequacy of end anchorage length must be checked during the design process to avoid premature failure of the system. 

In this section, provisions of an existing methodology for predicting end debonding (*fib* Bulletin 90 [10] and Zilch et al. [47]) are compared to experimental results to check its accuracy when different testing temperatures are used. According to the proposed method for the NSM technique, the end anchorage needs to be checked at the point where the FRP strip is first required for load-bearing purposes. This point, named *X*, has been assumed to be the one at which the moment equals the yielding moment of the unstrengthened control beam, considering the “shift rule”. At that point, the bond capacity of a FRP strip can be obtained as follows:

(1) $$F_{fbd}=\{\begin{array}{c} 0.95b_{f}\tau_{bld}\sqrt[{4}]{a_{r}}l_{b}\left(0.4-0.0015l_{b}\right)\;\;\;\;\;\;\;\;\;\;\;\;\;\;\;\;\;\;\;\;\;\;\;\;\;\;\;\;\;\;\;\;\;\;\;\;\;\;\;\;\;\;\;\;for\;l_{b}\leq 115\;\mathrm{mm}\;\\ 0.95b_{f}\tau_{bld}\sqrt[{4}]{a_{r}}\left[26.2+0.065\tan h\left(a_{r}/70\right)\left(l_{b}-115\right)\right]\;\;\;\;\;for\;l_{b}>115\;\mathrm{mm} \end{array}$$

where *F_fbd_* is the design bond capacity per strip in N, *b_f_* is the width of the strip in mm, *a_r_* is distance from the longitudinal axis of the strip to the free edge of the beam section in mm, and *l_b_* is the available anchorage length of the CFRP strip in mm. In addition, *τ_bld_* is the design bond strength of the NSM CFRP strips and can be assumed to be the minimum between the concrete bond strength and the adhesive bond strength as:

(2) $$\tau_{bld}=min\left(\alpha_{ba}\tau_{bak}\;,\;\alpha_{bc}\tau_{bck}\right)$$

where, *α_ba_* (ranging between 0.5 and 0.85) and *α_bc_* (ranging between 0.85 and 1) are product-specific factors for the long-term behavior of the adhesive and concrete, respectively. In this study, 0.85 and 1 were used as the values of *α_ba_* and *α_bc_*, respectively [10,47]. Finally, *τ_bak_* is the characteristic bond strength of the adhesive in MPa and *τ_bck_* is the characteristic bond strength of concrete according to:

(3) $$\tau_{bak}=k_{sys}\sqrt{\left[2f_{t,epoxy}-2\sqrt{f_{t,epoxy}^{2}+f_{c,epoxy}f_{t,epoxy}}+f_{c,epoxy}\right]f_{t,epoxy}}$$

(4) $$\tau_{bck}=k_{bck}\sqrt{f_{c}}$$

where *f_c_* is the compressive strength of the concrete in MPa (obtained from Table 2); *f_t,epoxy_* is the tensile strength of the adhesive in MPa (obtained from Table 4 for different temperatures); *f_c,epoxy_* is the compressive strength of the adhesive in MPa (obtained from Table 4 for different temperatures); and *k_sys_* (ranging between 0.6 and 1) and *k_bck_* (equal to 4.5) are the product-specific factors of the adhesive and concrete, respectively. In this study, a *k_sys_* value of 1 was used [10,47]. The effect of temperature on *f_c_* was considered based on *fib* Model code 2010 [48].

To verify the experimental failure modes, the aforementioned procedure was applied to the tested specimens. To this end, the experimental results in Table 5 (*P_y_*, *P_u_*, and *ε_y,FRP_*) were used to calculate the bond resistance of the CFRP-strengthened beams according to the following steps:

- Step 1: calculate the moment capacity of the unstrengthened beam at the yielding point of the steel reinforcement: *M_y_ = P_y_L*_1_/2, where *L*_1_ is shear span of the beam.
- Step 2: calculate the position of point *X* from the beam support, where the FRP strip is first required for load-bearing purposes: *X* = 2*M_y_/P_u_*.
- Step 3: calculate the actual strip force at a point corresponding to the yielding load of the control beam: *F_act_ = ε_y,FRP_ E_FRP_ A_FRP_*, where *ε_y,FRP_* is the CFRP strain at a level of load equal to the yielding load of the control beam of the same group, and *E_FRP_* and *A_FRP_* are CFRP modulus of elasticity and CFRP area, respectively.
- Step 4: calculate the available anchorage length considering the “shift rule”: *L_b_ = X* − *a* − 0.9*d(*cot*θ* − cot*α*)/2, where *a* is the distance between the support and end of the FRP strip, *θ* is the angle between the concrete compression strut and the beam axis perpendicular to the shear force, and *α* is the angle between the shear reinforcement and the beam axis perpendicular to the shear force. More information regarding to the “shift rule” can be found in Eurocode 2 [49].
- Step 5: calculate the design bond strength using Equation (2).
- Step 6: calculate the bond resistance of one CFRP strip using Equation (1) and multiply it by the number of CFRP strips.
- Step 7: calculate the ratio of actual strip force obtained from Step 3 to bond resistance obtained from Step 6 (*F_act_/F_fbd_*). If this ratio is greater than one, the actual force in the NSM strip is larger than the bond capacity predicted by Equation (1), indicating that the beam will fail due to premature end debonding. On the contrary, if the aforementioned ratio is less than one, the bond capacity would exceed the actual force in the NSM strip, indicating that the design is conservative and no end debonding would occur.

In this work, *L_1_* and *a* are equal to 750 mm and 125 mm, respectively (see Figure 1). Furthermore, *a_r_* is equal to 70, 46.5, and 35 mm for strengthened beams with one strip, two strips, and three strips, respectively.

Table 6 shows the intermediate results for predicting the end debonding phenomenon in the strengthened beams. According to Table 6, the ratio of *F_act_/F_fbd_* was less than one for specimens subjected to 40, 50, and 60 °C, which was in line with the experimental observation (FRP rupture without end debonding) which means the anchorage length was sufficient to transfer the actual strip force without premature end debonding. Nevertheless, further increases in the testing temperature up to 70 °C, resulted in a large reduction in the material properties of the adhesive and, subsequently, to larger strains (and forces) in the CFRP strip, which resulted in ineffective bonds between the FRP strip and the adhesive, thus leading to end debonding. This was confirmed by experimental observation, as the beam tested at 70 °C failed by end debonding. Finally, according to analytical predictions for the specimen at 85 °C, an end debonding failure mode was expected but the beam eventually failed by concrete crushing (as indicated previously, concrete strains attained typical values of ultimate strain). In view of these results, the comparison of analytical predictions and experimental results confirmed the accuracy of the proposed methodology. However, more experimental work is needed to evaluate the adequacy of the aforementioned formulation for higher testing temperatures. 

**Figure 11:**
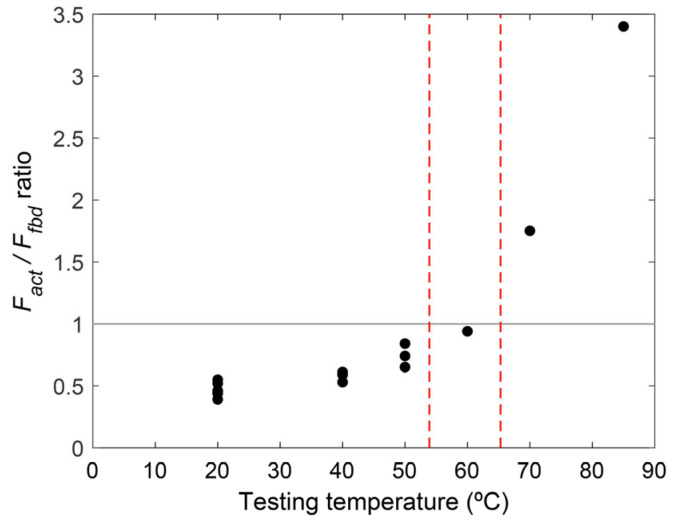
Evolution of the *F_act_/F_fbd_* ratio with respect to testing temperature (vertical dash lines indicate the range of the *T_g_* of the epoxy).

The evolution of the *F_act_/F_fbd_* ratio with respect to testing temperature is shown in Figure 11. According to this plot, this ratio tended to remain constant for testing temperatures below the *T_g_* of the epoxy adhesive. Once testing temperature approached the lower limit of the range of values for the *T_g_*, the ratio started to increase to a value near to 1 for testing temperatures close to the mid position of the range of values of *T_g_* (i.e., at 60 °C, the ratio *F_act_/F_fbd_* equals 0.94). Finally, when the testing temperature overtook the upper limit of the range of values for *T_g_*, the *F_act_/F_fbd_* ratio rapidly increased. This demonstrates the effect of high service temperatures on the bond capacity of the adhesive joint, which, in the end, affects the failure mode of NSM CFRP-strengthened RC beams.

### 3.4. Limitations

The analytical predictions on end debonding phenomena presented in Figure 11 are limited to the presented experimental database. In this sense, a more detailed analysis should be performed on a larger experimental data base, which includes a larger number of specimens tested under higher service temperatures (beyond the *T_g_* of the epoxy adhesive), as this may affect the bond performance. In addition, concrete compressive strength is also an important parameter in the proposed methodology. Therefore, the expanded database should also include specimens with different ranges of concrete compressive strength.

## 4. Conclusions

In this work, an experimental program designed to evaluate the effect of high service temperatures on the flexural performance of NSM CFRP-strengthened RC beams was presented. The results in terms of load–deflection curves and failure modes are presented and discussed. Furthermore, an analytical procedure in the literature [10,47] was applied to predict the occurrence of the end debonding failure mode in NSM CFRP-strengthened RC beams. Based on the experimental and analytical results, the following conclusions can be drawn:

1. The obtained experimental results showed a good performance of the NSM technique to strengthen concrete structures for temperatures relatively close to the epoxy *T_g_*.
2. The capacity (ultimate load) of the strengthened specimens was not affected by testing temperatures equal to 40, 50, and 60 °C, whilst the application of testing temperatures higher than the *T_g_* of the epoxy resulted in a reduction in beam capacity equal to 3.95% and 10.45% for beams SB2S-2-70 and SB2S-2-85, respectively.
3. In all groups of specimens, the control (unstrengthened) beams failed by concrete crushing after yielding of the steel reinforcement. The application of different testing temperatures showed that strengthened beams subjected to 20, 40, 50, and 60 °C (near and in the lower range of the *T_g_* of the epoxy) failed by FRP rupture, whilst the failure mode changed when the testing temperature was beyond the upper range of the *T_g_* of the epoxy adhesive (i.e., 70 °C). This is evidence of the effect of temperature on the performance of the adhesive and the bond resistance between the adhesive and concrete and, therefore, on the failure mode of the strengthened flexural element.
4. Analytical predictions on premature end debonding were compared to the experimental results and good agreement was found, thus confirming the capability of the analytical procedure to predict end debonding failure.
5. The analytical procedure demonstrated that proneness to premature end debonding of the NSM joint is more pronounced for testing temperatures near the *T_g_* of the epoxy adhesive, showing that temperature can be a key factor if the *T_g_* of the epoxy adhesive is exceeded.

## Figures and Tables

**Table 1:** Testing matrix.

Experimental Group	Beam ID	Testing Temperature (°C)	Steel Reinforcement Ratio, *ρ* (%)	Concrete Compressive Strength, *f_c_* (MPa)	No. of Strips	CFRP Area (mm^2^)	CFRP Ratio (%)
Group 1	CB-1-20	20	1.14	31.8	-	-	-
	CB-1-40	40			-	-	-
	SB1S-1-20	20			1	14	0.06
	SB1S-1-40	40			1	14	0.06
	SB2S-1-20	20			2	28	0.12
	SB2S-1-40	40			2	28	0.12
	SB3S-1-20	20			3	42	0.18
	SB3S-1-40	40			3	42	0.18
Group 2	CB-2-20	20	1.14	40.8	-	-	-
	CB-2-70	70			-	-	-
	SB2S-2-20	20			2	28	0.12
	SB2S-2-60	60			2	28	0.12
	SB2S-2-70	70			2	28	0.12
	SB2S-2-85	85			2	28	0.12
Group 3	CB-3-20	20	0.79	48.1	-	-	-
	CB-3-50	50			-	-	-
	SB1S-3-20	20			1	14	0.06
	SB1S-3-50	50			1	14	0.06
	SB3S-3-20	20			3	42	0.18
	SB3S-3-50	50			3	42	0.18
Group 4	CB-4-20	20	1.14	48.1	-	-	-
	CB-4-50	50			-	-	-
	SB3S-4-20	20			3	42	0.18
	SB3S-4-50	50			3	42	0.18

**Table 2:** Details of concrete batches.

Batch Number	Group	Cement Type	Cement Content (kg/m^3^)	w/c ^a^ Ratio	Concrete Age (Days)	Compressive Strength, *f_c_* (MPa)	Tensile Strength, *f_t_* (MPa)	Modulus of Elasticity, *E_c_* (GPa)
1	Group 1	I-42.5R	390	0.46	36	31.8 (6.6%) ^b^	4.2 (2.3%) ^b^	31.5 (7.8%) ^b^
2	Group 2		333	0.48	37	40.8 (2.8%) ^b^	5.2 (0.5%) ^b^	29.4 (0.8%) ^b^
3	Group 3, 4		390	0.41	90	48.1 (2.3%) ^b^	3.7 (6.0%) ^b^	41.2 (5.3%) ^b^

^a^ water to cement ratio. ^b^ Coefficient of variation (CoV) is indicated in brackets.

**Table 3:** Steel reinforcement mechanical properties.

Group	Yielding Stress, *f_y_* (MPa)	Ultimate Stress, *f_u_* (MPa)	Modulus of Elasticity, *E_s_* (GPa)
Group 1, 2	573.2 (1.1%) ^a^	673.9 (0.8%) ^a^	200.8 (0.7%) ^a^
Group 3, 4	586.4 (2.3%) ^a^	707.7 (1.7%) ^a^	205.1 (1.0%) ^a^

^a^ Coefficient of variation (CoV) indicated in brackets.

**Table 4:** Mechanical properties of epoxy adhesive tested at different temperatures [8].

Testing Temperature (°C)	Tensile Strength, *f_t,epoxy_* (MPa)	Tensile Modulus of Elasticity, *E_epoxy_* (MPa)	Compressive Strength, *f_c,epoxy_* (MPa)
20	28.0 (0.4%) ^a^	8102.4 (0.8%) ^a^	79.9 (0.1%) ^a^
40	23.0 (0.9%) ^a^	5520.8 (3.2%) ^a^	60.5 (4.3%) ^a^
50	19.6 (3.6%) ^a^	4289.6 (2.3%) ^a^	49.0 (1.2%) ^a^
60	6.9 (2.9%) ^a^	673.2 (3.6%) ^a^	44.2 (2.3%) ^a^
70	2.7 (3.7%) ^a^	271.5 (4.8%) ^a^	24.4 (0.4%) ^a^
85	1.9 (0.0%) ^a^	247.5 (0.7%) ^a^	9.9 (7.0%) ^a^

^a^ Coefficient of variation (CoV) is indicated in brackets.

**Table 5:** Experimental results of tested beams.

Group	Beam ID	Cracking Load, *P_cr_* (kN)	Yielding Load, *P_y_* (kN)	Ultimate Load, *P_u_* (kN)	CFRP Strain at Yielding ^a^, *ε_y,FRP_* (mm/mm)	Ultimate CFRP Strain, *ε_u,FRP_* (mm/mm)	Strength Increase Ratio ^b^	Failure Mode
Group 1	CB-1-20	6.53	39.90	41.87	-	-	-	CC
	CB-1-40	5.51	39.68	43.39	-	-	-	CC
	SB1S-1-20	7.11	44.90	53.44	0.0038	0.0139	1.28	FR
	SB1S-1-40	6.07	44.69	53.63	0.0040	0.0142	1.28	FR
	SB2S-1-20	7.21	49.40	66.50	0.0033	0.0132	1.59	FR
	SB2S-1-40	6.12	49.27	64.06	0.0035	0.0134	1.53	FR
	SB3S-1-20	7.56	56.80	77.04	0.0032	0.0127	1.84	FR
	SB3S-1-40	6.41	55.60	76.77	0.0028	0.0124	1.83	FR
Group 2	CB-2-20	3.69	38.59	41.71	-	-	-	CC
	CB-2-70	2.12	37.76	40.26	-	-	-	CC
	SB2S-2-20	4.79	48.21	63.36	0.0032	0.0131	1.52	FR
	SB2S-2-60	2.09	47.88	63.39	0.0030	0.0119	1.52	FR
	SB2S-2-70	1.82	46.70	60.86	0.0028	0.0103	1.46	ED
	SB2S-2-85	1.68	46.47	56.72	0.0027	0.0100	1.36	CC
Group 3	CB-3-20	7.43	30.75	34.28	-	-	-	CC
	CB-3-50	7.00	30.00	33.57	-	-	-	CC
	SB1S-3-20	7.82	36.88	45.50	0.0042	0.0148	1.33	FR
	SB1S-3-50	6.00	35.36	45.15	0.0037	0.0134	1.32	FR
	SB3S-3-20	8.85	46.50	71.40	0.0029	0.0138	2.08	FR
	SB3S-3-50	6.75	45.25	70.00	0.0028	0.0125	2.04	FR
Group 4	CB-4-20	6.70	37.89	41.84	-	-	-	CC
	CB-4-50	5.95	38.71	42.10	-	-	-	CC
	SB3S-4-20	9.60	53.36	75.10	0.0026	0.0123	1.79	FR
	SB3S-4-50	8.20	51.00	77.68	0.0028	0.0132	1.86	FR

Note: CC = concrete crushing after steel yielding; FR = FRP rupture; ED = end debonding. ^a^ CFRP strain at a level of load equal to the yielding load of the control beam of the same group. ^b^ Ratio of ultimate load of strengthened beam to ultimate load of the control unstrengthened beam of the same group (tested at 20 °C).

**Table 6:** End debonding predictions according to *fib* bulletin 90 (*fib* 2019).

Beam ID	Step 1	Step 2	Step 3	Step 4	Step 5			Step 6	Step 7
	*M_y_* (kN.mm)	*X* (mm)	*F_act_* (kN)	*l_b_* (mm)	*τ_bak_* (MPa)	*τ_bck_* (MPa)	min (*τ_bak,_ τ_bck_*) (MPa)	*F_fbd_* (kN)	*F_act_/F_fbd_*
SB1S-1-20	14,962.5	560.0	9.0	328.3	22.9	21.6	21.6	21.8	0.39
SB1S-1-40	14,880.0	554.9	9.4	323.2	17.7	20.9	17.7	17.8	0.53
SB2S-1-20	14,962.5	450.0	15.7	218.3	22.9	21.6	21.6	32.2	0.46
SB2S-1-40	14,880.0	464.6	16.4	232.9	17.7	20.9	17.7	26.9	0.61
SB3S-1-20	14,962.5	388.4	22.8	156.7	22.9	21.6	21.6	41.0	0.52
SB3S-1-40	14,880.0	387.7	20.0	155.9	17.7	20.9	17.7	33.7	0.59
SB2S-2-20	14,471.3	456.8	15.0	225.1	22.9	24.4	22.9	34.5	0.44
SB2S-2-60	14,160.0	446.8	14.1	215.0	10.1	22.9	10.1	15.0	0.94
SB2S-2-70	14,160.0	465.3	13.2	233.6	5.0	22.5	5.0	7.6	1.75
SB2S-2-85	14,160.0	499.3	13.0	267.6	2.4	21.9	2.4	3.8	3.40
SB1S-3-20	11,531.3	506.9	9.9	275.2	22.9	26.5	22.9	21.5	0.46
SB1S-3-50	11,250.0	498.3	8.8	266.6	14.5	25.3	14.5	13.4	0.65
SB3S-3-20	11,531.3	323.0	20.9	91.3	22.9	26.5	22.9	38.2	0.55
SB3S-3-50	11,250.0	321.4	20.0	89.7	14.5	25.3	14.5	23.9	0.84
SB3S-4-20	14,208.8	378.4	18.9	146.7	22.9	26.5	22.9	43.1	0.44
SB3S-4-50	14,516.3	373.7	20.2	142.0	14.5	25.3	14.5	27.2	0.74

## Data Availability

Not applicable.
